# Dimensionality Reduction in Surrogate Modeling: A Review of Combined Methods

**DOI:** 10.1007/s41019-022-00193-5

**Published:** 2022-08-21

**Authors:** Chun Kit Jeffery Hou, Kamran Behdinan

**Affiliations:** Department of Mechanical and Industrial Engineering, University of Toronto, Toronto, ON Canada

**Keywords:** Dimensionality reduction, Surrogate modeling, Machine learning, High dimensionality, Neural networks

## Abstract

Surrogate modeling has been popularized as an alternative to full-scale models in complex engineering processes such as manufacturing and computer-assisted engineering. The modeling demand exponentially increases with complexity and number of system parameters, which consequently requires higher-dimensional engineering solving techniques. This is known as the curse of dimensionality. Surrogate models are commonly used to replace costly computational simulations and modeling of complex geometries. However, an ongoing challenge is to reduce execution and memory consumption of high-complexity processes, which often exhibit nonlinear phenomena. Dimensionality reduction algorithms have been employed for feature extraction, selection, and elimination for simplifying surrogate models of high-dimensional problems. By applying dimensionality reduction to surrogate models, less computation is required to generate surrogate model parts while retaining sufficient representation accuracy of the full process. This paper aims to review the current literature on dimensionality reduction integrated with surrogate modeling methods. A review of the current state-of-the-art dimensionality reduction and surrogate modeling methods is introduced with a discussion of their mathematical implications, applications, and limitations. Finally, current studies that combine the two topics are discussed and avenues of further research are presented.

## Introduction

Data mining has become a rapidly growing field in recent years. At the same time, data generation has seen a surge in volume, leading to a growth in size, complexity, and data dimensionality. High-dimensional data exists where the number of data features is on the order of the number of samples or observations [1]. These datasets can be computationally expensive to learn and generating mapping functions between input and output can be a cumbersome task. Thus, reducing the number of features, or the problem dimensionality, can greatly simplify the learning and training of regression and classification models for extracting patterns in the data. Dimensionality reduction (DR) techniques seek to reduce the data dimensionality and identify intrinsic data structure while sacrificing minimal accuracy and information. DR can be achieved through feature elimination, feature selection, or feature extraction. Feature elimination involves reducing the input dimension space by eliminating features of the dataset that are deemed unimportant. Although this simplifies the computations afterwards, no information is gained by dropping those features. Feature selection involves using statistics to determine and rank features based on their information contribution to the overall dataset. These methods can be categorized as filter and wrapper methods and have been explored in detail in [2]. It is important to note that there is no universal method for ranking data features as different tests will yield different contribution scores. Global sensitivity analysis methods [3], which identify the ‘most important’ inputs in unstructured datasets and ignore the others, have emerged as a novel feature selection method for machine learning (ML) prediction models [4]. Finally, feature extraction methods, like principal component analysis (PCA), create new independent features that are combinations of the original dataset features.

In this paper, dimensionality reduction methods are classified as linear and non-linear methods. Linear DR methods transform data to a lower-dimension feature space through linear kernelization and combinations of original variables. The linear techniques presented in this paper predominantly perform dimensionality reduction through linear algebra. Non-linear DR methods are applied when the initial data space contains nonlinear relationships and structure. These include kernel PCA (kPCA), manifold learning methods, and autoencoders. Typically, non-linear DR techniques generate a lower-dimensional representation of the data while preserving distance between data points. Furthermore, these methods can subsequently be supervised or unsupervised schemes. Unsupervised DR methods, such as PCA, only consider the input feature matrix for pattern identification, while supervised methods such as partial least squares (PLS) and linear discriminant analysis (LDA) consider both features and the responses. The overall goal of DR methods is to enhance the accuracy and efficiency of data mining by reducing the dataset and increasing data quality.

Surrogate models, or metamodels, approximate high-fidelity models using statistical methods without compromising on model accuracy or representation. These models can be used as analytical tools for model simplification [5], uncertainty quantification, or reducing ill-conditioning of optimization problems through incorporation of gradient information [6]. In the context of surrogate optimization models, local surrogates are updated within an iterative framework, while global surrogates are fitted only once with the training set [7]. The benefit of reduced runtimes and computational efficiency allows surrogates to be designed for real-time decision support environments and investigation of structural model uncertainty by simulating other model structures [8]. Typically, the models are trained with a set of in and output data and validated to emulate the full-scale simulations at much less computational cost [9]. Surrogate models can be categorized as interpolating and non-interpolating methods. Interpolating method such as Kriging rely on sufficient data around the points to be predicted. However, non-interpolation methods generate predictions without the need for data within the range of predicted values. For instance, neural networks (NNs) and support vector machines (SVMs) find the input–output mapping by minimizing error functions. Each category of non-interpolation methods composes of data-driven, multi-fidelity, and projection-based methods with corresponding linear and nonlinear prediction schemes. Due to the variety of engineering problems that surrogate models can be applied, selecting the appropriate model to implement typically depends on the available dataset size and the number of parameters.

**Fig. 1:**
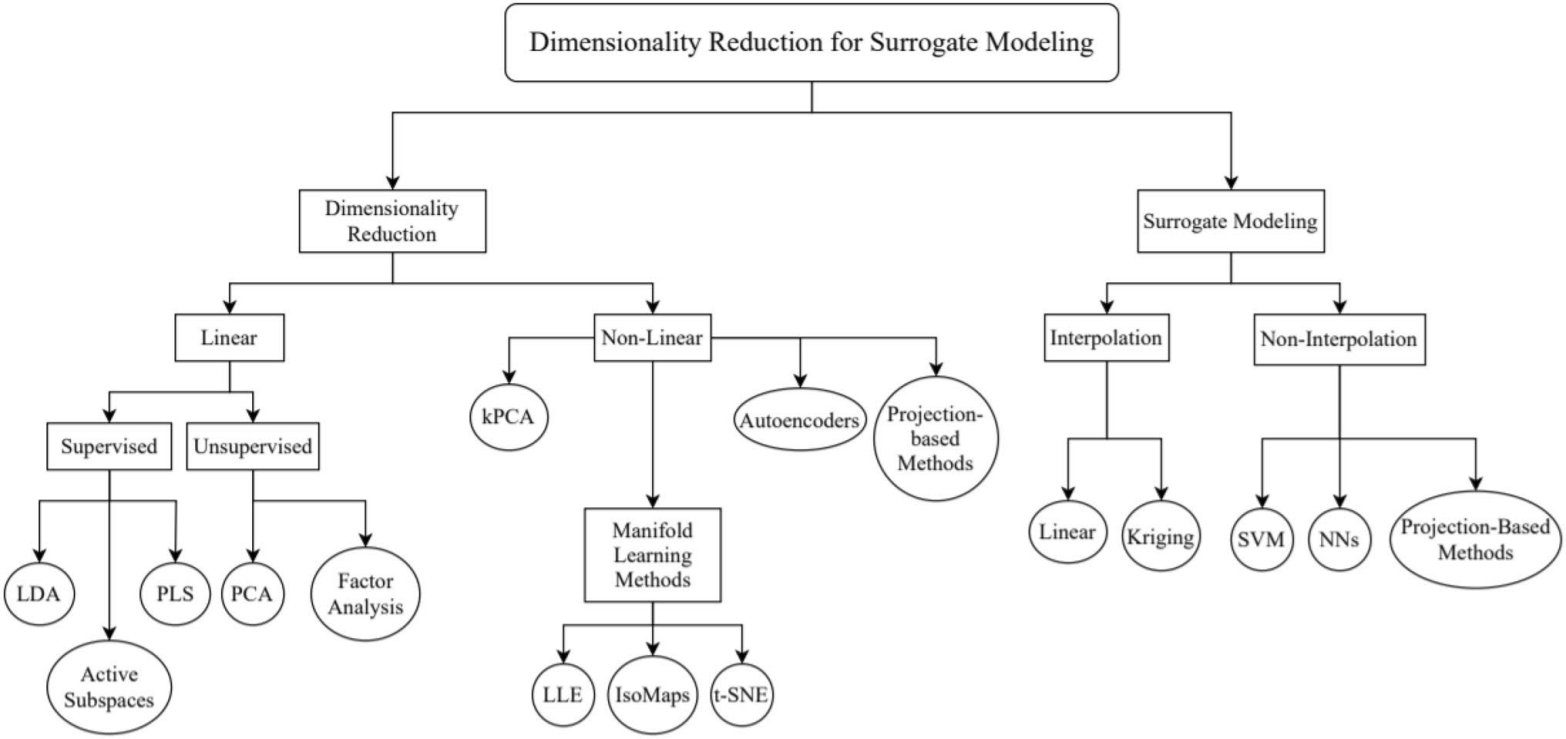
Overview of Dimensionality Reduction in Surrogate Modeling

The organization of this review paper is shown in Fig. 1. A comprehensive review of the current literature on dimensionality reduction and surrogate modeling methods is presented. As well as summarizing their mathematical interpretations and limitations, we investigate the current state-of-the-art applications of each and the potential for improving computational efficiency in fields where they have not been applied. We further investigate existing research efforts that combine dimensionality reduction with surrogate modeling (DRSM) and the contributions and needs for further research in this growing field of ML application.

## Linear Dimensionality Reduction

Dimensionality reduction aims to transform data in high-dimensional space into low-dimensional representations through feature extraction, elimination, or selection. In this section, we discuss several DR methods which utilize linear algebra relations to reduce the size of input data and associated applications of each method in the literature.

### Unsupervised Linear Methods

Unsupervised Linear dimensionality reduction techniques (PCA, KPCA, Factor Analysis) are employed at the pre-processing stage for surrogate modeling. These methods are trained using unlabeled data and only consider input features for discovering lower-dimensional representations.

#### Principal Component Analysis

Principal Component Analysis (PCA) is the most popular unsupervised learning method for linear dimensionality reduction.

The method considers *n* input samples with *k* features, excluding the output labels, and normalizes the dataset for comparable values across features. The covariance matrix, *R*, is computed for the input data and singular value decomposition is used to produce a diagonal matrix of principal component eigenvalues, *Ʌ*, and associated eigenvectors, *A,* as shown in (1).

1 $$R = A\Lambda \; A^{T}$$

The elements of the covariance matrix indicate the correlation of each component with corresponding input variables. The eigenvalues are correlated with the level of variance captured by the associated principal component’s eigenvector. The resulting principal components are linear expressions of the initial input data and are independent of one another. Taking advantage of the diagonal properties of the eigenvector matrix, *R* can be rewritten in terms of the principal component loading matrix, *L,* with dimensionality *r x k,* where *r* < *k,* as shown in (2).

2 $$R = LL^{T}$$

The principal components can be determined by multiplying the input data matrix by the loading matrix, as shown in (3).

3 $$P = XL$$

The number of retained *r* components is selected based on captured variance of the full data. The principal component matrix is multiplied with the loading matrix, which reconstructs the input features to *n x r* dimensions, as shown by (4).

4 $$\hat{X} = PL^{T}$$

**Fig. 2:**
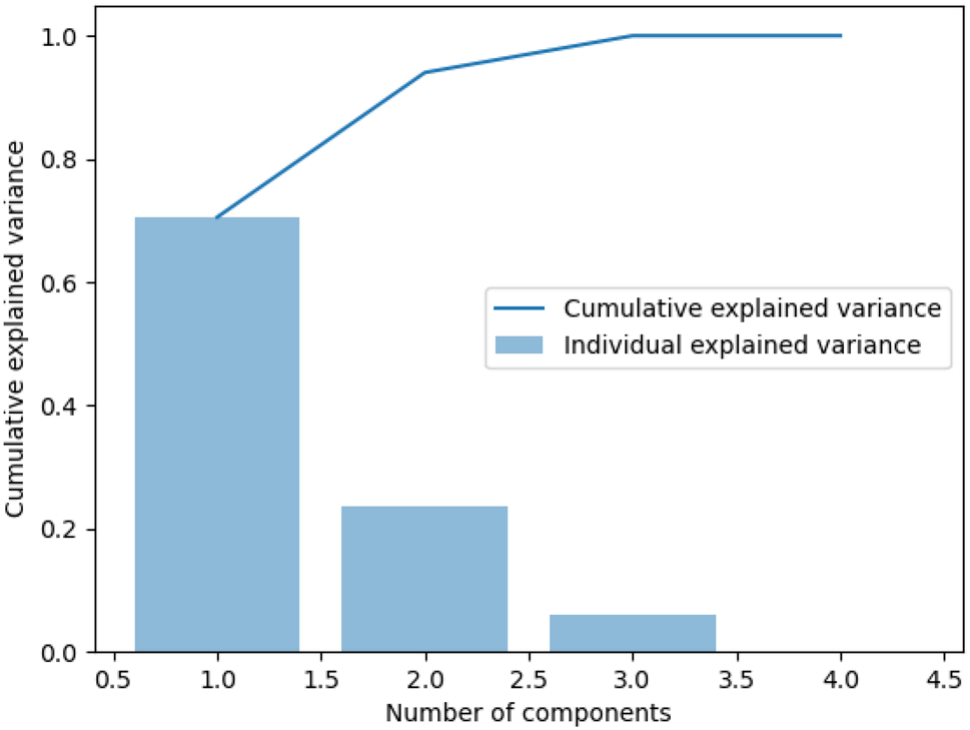
Variance v.s. Principal Components. Cumulative variance line and contributed variance by each subsequent principal component as bars

Figure 2 shows the variance captured by each principal component. The curve indicates the cumulative variance that is captured with each additional principal component, while the bars show the contributed variance by each principal component.

PCA has been applied to a variety of engineering simulation problems to reduce complexity, ease computations, and extract key information for simplified reconstruction of datasets with quantitative variables. For instance, [10] utilized principal component analysis for feature extraction in nonlinear FEA U-structure. 24 input parameters were converted to 11 principal components, which captured 98.5% of the total variance.

PCA has also drastically improved FEA procedures for biomechanics. Grassi et al. [11] developed a PCA-based statistical finite element modeling method for reconstructing bone shape and density. Due to the shortage of available computed tomography-based FE models, bone shape and density indexation through PCA were used to reproduce a lower-order model for obtaining biomechanical properties of bones. The statistical model was based on collected femoral CT datasets and the shape pre-processing was based on isotopological meshes. After scaling the meshes, PCA was applied to both the bone shape and mineral density (BMD) properties. The number of components maintained for shape reconstruction was based on the database mean reconstruction error compared to the CT data’s resolution, while a relative error threshold was used for BMD components. Upon validating with leave-one-out tests, the proposed PCA-based statistical model reconstructed both the bone shape and mineral density properties within the tolerated error thresholds. However, coupling between shape and mineral density variations was not considered in the linear statistical model, which highlights the need for non-linear methods to solve high complexity FEA. Pellicer-Valero et al. [12] employed ML techniques for simulating the biomechanical behavior of the human liver under applied load. Liver images were processed in MATLAB and the loads were applied in increments to aid with convergence under large deformations and eliminate viscoelastic effects. PCA was applied to the vector of liver geometry inputs and a feedforward NN and random forest regressor were compared with literature methods for developing the non-linear mapping. While the proposed algorithm successfully predicted liver geometry under arbitrary loads, the approximation can be further improved by using convolutional layers in the image segmentation task prior to training the neural network.

#### Factor Analysis

Alternatively, factor analysis (FA) is an unsupervised dimensionality reduction technique for determining the relationships within a set of random variables observed or measured for each datapoint of a group [13]. The method aims to identify the number of factors and the underlying structure of a set of variables for dimensionality reduction [14, 15]. Factors, or latent variables, are presumed to exist in the observed dataset and cannot be measured or observed directly. FA can be interpreted as an extension of PCA, as both methods share similar assumptions regarding normal distributed variables and bivariate distribution of variable pairs [16]. While PCA decomposes the correlation matrix of continuous variables into principal components expressed as linear functions of the original set of feature variables, FA decomposes a correlation matrix adjusted for unique factors in the diagonal. Furthermore, PCA assumes the total variance is equivalent to common variance, while FA assumes unique variance also contributes to the total variance. The FA variables are expressed as linear combinations of common and unique factors. Unique factors are explained as unreliability due to measurement error and data variation and are unique for each variable, while common factors are shared among different variables. FA reduces the number of variables through factor extraction and factor rotation. In factor extraction, the type of model and number of factors to extract is selected. Then, the matrix of extract factors is rotated using orthogonal or oblique rotations for improving interpretability of results.

#### Mixed Data Type Methods

Real datasets often contain mixed-type variables such as continuous, binary, and categorical sets. Since linear PCA minimizes the squared loss function to determine principal components, the method may not be appropriate for data that is discontinuous [17]. PCA methods for mixed data types have been implemented in several R packages such as PCAmixdata [18], FactoMineR [19, 20], and ade4 [21]. FactoMineR uses an extended method of the FA method (FAMD) based on findings by [22, 23]. Pagès [24] further developed the FAMD method for survey analysis, ecology, and time series problems. The FAMD method works as PCA for quantitative variables and uses multiple correspondence analysis (MCA) for categorical and qualitative variables. Overall, the factors are determined based on maximizing the sum of correlations in qualitative and quantitative variables and selecting them based on highest squared correlation coefficients.

### Supervised Linear Methods

Linear Discriminant Analysis (LDA), Partial Least Squares (PLS), and active subspace methods are supervised, linear alternatives to PCA. As previously mentioned, these methods are trained using labelled input and output data. In the context of reducing dimensionality for surrogate models, supervised methods produce more suitable topology representations of input–output maps compared to unsupervised methods [25].

#### Linear Discriminant Analysis

Linear discriminant analysis (LDA), derived as an extension from the general form of the Fisher’s discriminant analysis, is a feature selection method which performs the dimensionality reduction through maximizing class-separation distance and minimizing distance within class data (the features). The underlying assumptions of LDA include normally distributed data behavior and homogeneous variance among variables. While PCA works better for smaller datasets, LDA is superior for multi-class classification problems and dimensionality reduction for classification in later steps. LDA performs best for Gaussian classes with identical covariance and normally distributed datasets, as a solution that minimizes the expected error. The method can perform well so long as the data is close to normality behavior.

In the LDA procedure, the mean vectors, *m,* of each data class are computed first. The within-class ${S}_{W}$ and between-class ${S}_{B}$ scatter matrices are computed through (5) and (6), where *m* is the overall mean, *n* is the number of classes, and ${m}_{i}$ and ${N}_{i}$ denote the sample mean and sizes, respectively.

5 $$S_{B} = \mathop \sum \limits_{i = 1}^{c} N_{i} \left( {m_{i} - m} \right)\left( {m_{i} - m} \right)^{T}$$

6 $$S_{W} = \mathop \sum \limits_{i = 1}^{c} \mathop \sum \limits_{{x \in D_{i} }}^{n} \left( {x - m_{i} } \right)\left( {x - m_{i} } \right)^{T}$$

Next, eigendecomposition is performed to obtain the linear discriminants through (7), where *λ* and *v* are the eigenvalue and eigenvector matrices.

7 $$S_{W}^{ - 1} S_{B} v = \lambda v$$

Finally, the number of linear discriminants for transforming the dataset into the lower-dimensional feature space follows a similar procedure to the latter steps of Fig. 2.

#### Partial Least Squares

The Partial Least Squares (PLS) method is a dimensionality reduction method commonly used in multivariate calibration and classification problems [26, 27]. Unlike PCA which calculates hyperplanes for maximum variance in the predictors/input data, PLS projects the input and response/output variables to a new feature space based on maximum covariances and finds a regression mapping function relating them. PLS regression reduces the dimensionality by fitting multiple response variables into a single model. This is analogous to a multilayer perceptron, where the hidden layer nodes can be determined by the number of retained PLS components after applying the reduction [28]. Furthermore, the multivariate consideration does not assume fixed predictors. Thus, errors associated with each predictor can be considered, which makes PLS robust in measuring uncertainty. Equations (8) and (9) define the underlying PLS model.

8 $$X = AC^{T} + E$$

9 $$Y = BD^{T} + F$$

Here, *X* and *Y* are the non-reduced predictors and responses, respectively. *A* and *B* are the projections of *X* and *Y*, *C* and *D* are the respective orthogonal loading matrices, and *E* and *F* are error terms. In the PLS algorithm, the covariance of *A* and *B* is maximized when projecting *X* and *Y* into the new feature space.

PLS is applied to datasets with multicollinear predictors, and in problems with limited training data and high dimensionality such as bioinformatics and neuroscience. However, PLS tends to perform poorly when screening factors that have minimal effect on the response and general calculations are slower than traditional multivariate methods [29].

#### Active Subspace Methods

Active subspace methods have also been used for detecting directions of strongest variability in a function to construct a low-dimensional subspace of the function’s uncertain inputs [30], 31]. This is analogous to PCA, except gradient information is considered in the eigendecomposition. The effective variability in the model’s output due to uncertain inputs is captured in the directions of the active subspace. Given a set of *N* uncertain data points with model inputs *X* and outputs *f*, the output gradients with respect to the input parameters are used to construct the active subspace directions matrix, denoted by *C,* given by (10).

10 $$C = \mathop \smallint \limits_{\Omega }^{{}} \left( {\nabla_{\varepsilon } f} \right)\left( {\nabla_{\varepsilon } f} \right)^{T} \mu \left( {d\varepsilon } \right)$$

Here,$\varepsilon$ denotes the canonical variables mapped to a given mathematical model in the domain $\Omega$ and $\upmu$ represents the probability density function. *C* is a symmetric and positive definite matrix, which can be expressed as a spectral decomposition of eigenvalue $\Lambda$ and orthonormal eigenvector $W$ matrices, as shown in (11). The input vector is transformed into the active space by (12) and the approximated regression surface *G(y)* is computed in this reduced space by (13).

11 $$C = W\Lambda W^{T}$$

12 $$y = W^{T} X$$

13 $$G\left( y \right) = f\left( {Wy} \right) = f$$

Overall, active subspace methods are accurate for classes of functions with decay in the eigenvalues of *C* and change predominantly in low-dimensional input subspaces. However, the gradient information must be available for active subspaces and must be approximated, otherwise, these methods perform poorly [32].

While linear dimensionality methods have successfully reduced complexity in classification problems and uncertainty analysis, these methods are limited to datasets that are linearly separable. In the case of inseparable data, nonlinear techniques must be considered such as Kernel PCA and autoencoders.

## Nonlinear Dimensionality Reduction

In this section, nonlinear dimensionality reduction methods are presented with a fundamental description of the mathematical interpretations and limitations of each. Many non-linear dimensionality reduction methods have been developed as extensions of linear methods, such as kPCA and non-linear PLS. Furthermore, methods based on layered manifolds and artificial neural networks (ANNs) have been developed for understanding non-linear behavior in datasets and generating lower-dimensional mappings between input and output. Only unsupervised methods are presented in the following section, as these are commonly integrated with surrogate modeling methods in the literature. Supervised non-linear dimensionality reduction methods have been developed in [33], [34], but will not be discussed in detail.

### Kernel Principal Component Analysis

Kernel PCA (kPCA) is a classical approach for nonlinear dimensionality reduction. The method projects the linearly inseparable data into a higher dimensional Hilbert space $k\left({x}_{i},{x}_{j}\right)$ through a kernel function φ, as shown by (14), where PCA method is performed afterwards.

14 $$k\left( {x_{i} ,x_{j} } \right) = \varphi \left( {x_{i} } \right)\varphi \left( {x_{j} } \right)^{T}$$

The kPCA method is more commonly used for classification problems, such as face recognition, and is more computationally expensive to perform than linear PCA. Gonzalez et al. [35] combined kPCA with the Proper Generalized Decomposition method for acquiring parametric solutions of liver geometry. kPCA was used to extract information on hidden parameters such as shape and microstructure behavior. Wang [36] investigated the use of combining kPCA with active shape models (ASM) for facial recognition. They used ASMs to represent the deformation patterns of an object’s shape and to locate the object in new images. Furthermore, a Gaussian kernel was determined to be on the order of the nearest neighbor distance between two data points. This ensured maximum separation in the resulting kPCA feature space. The combined kPCA-ASM method achieved lower classification error rates and succeeded in revealing complicated structures in data that could not be achieved with standard ASMs. The challenge with kPCA remains in the difficult kernel selection task that is problem-dependent.

### Non-linear Partial Least Squares Methods

Several nonlinear versions of PLS have been discussed in the literature [37]. The concept of nonlinear PLS modeling methods is divided into two representative models. The first model applies a nonlinear transformation to the sample data and a linear model is constructed as the new representation [38, 39]. To overcome the lack of interpretability with these models, the second set of methods assumes nonlinearity in the latent variables and the extracted latent vectors remain as linear combinations of the original variables rather than a transformation [40–42]. For instance, [40] proposed a quadratic, error-based algorithm for updating weights in the PLS method to incorporate nonlinearities. To relax the assumption of a linear relationship between latent components and the response, the authors introduced additional loops for iterating between latent components and the response in the algorithm.

### Multidimensional Scaling

Multidimensional scaling (MDS) methods are multivariate data analysis techniques used for translating high-dimensional objects into low-dimensional representations of mapped points. The mapping is established such that the distances between objects are preserved as well as possible since there are some dissimilarities between the spaces. Classical MDS takes an input matrix of dissimilarities between items and outputs and seeks lower-dimensional representation by minimizing the difference of similarities in input and embedded spaces [43] shown in (15). Here, *x* denotes the data in the higher dimension and *y* denotes the embedded data.

15 $$c = \mathop \sum \limits_{i = 1}^{n} \mathop \sum \limits_{j = 1}^{n} \left( {x_{i}^{T} x_{j} - y_{i}^{T} y_{j} } \right)^{2}$$

The classical MDS method closely resembles that of PCA but differs in the matrix output from the eigendecomposition. The number of retained components is determined based on the *m* largest eigenvalues corresponding to eigenvectors from singular value decomposition of the cost function to obtain the Gram matrix. According to [43], the classical MDS method uses a linear kernel for optimization, hence it is a linear DR method. However, depending on the interpretation of the input matrix and dissimilarities’ relation to point distances, MDS algorithms optimize different variations of the Euclidean cost functions. In cases where distances are preserved rather than similarities of points, these cases of MDS become non-linear algorithms which optimize the stress error function *c* shown in (16) and (17). Here, *g(x)* denotes the monotonic, non-linear transformation of pairwise distances.

16 $$c = \mathop \sum \limits_{i = 1}^{n} \mathop \sum \limits_{j = 1}^{n} \left( {||x_{i} - x_{j} || - ||y_{i} - y_{j} ||} \right)^{2}$$

17 $$c = \mathop \sum \limits_{i = 1}^{n} \mathop \sum \limits_{j = 1}^{n} \left( {g(x) - ||y_{i} - y_{j} ||} \right)^{2}$$

Specifically, metric MDS iteratively optimizes (16) where dissimilarities are proportional to distances, while nonmetric MDS optimizes (17) in cases containing ordinal dissimilarities where the rank order of distances must be close to the rank of dissimilarities. In both cases, the distance matrix in reduced space is compared against the high-dimensional distances and the coordinate positions in the lower dimensional space are corrected to minimize the stress function. Overall, the MDS methods yield very precise solutions with little computational cost. However, both metric and nonmetric MDS have their respective limitations. Metric MDS may lead to poor results if the interval scale condition is not met by the dataset being analyzed and nonmetric MDS may identify local minima as the ideal solution [44].

### Autoencoders

Autoencoders are unsupervised nonlinear dimensionality reduction methods with a NN structure whose output has the same shape as its input [45]. The architecture of the autoencoder can be convolutional (CNN), recursive (RNN) [46], or a traditional multilayer perceptron. Several studies have applied each NN structure for dimensionality reduction. Saenz et al. [47] used convolutional autoencoders for dimensionality reduction of climate data models. The autoencoder was compared against PCA and showed improved reconstruction of temperature fields based on the original dataset. The authors mention the potential for autoencoder-based DR methods to be applied for creating surrogate climate models in future works.

CNNs have been used to restore spatial features during image processing [48] and the neurons are connected to a small region of the previous layer, sharing their weights. This allows the system to adaptively learn spatial patterns in the input images and reduce the number of parameters to avoid overfitting [49, 50]. Stacked autoencoders have been used for improving computational efficiency and preserving spatial features in the original input space. Convolutional autoencoders have been used for extracting feature spatial features in geological models [51, 52, 53] and reducing data dimensionality for reconstructing images and classification tasks [54].

**Fig. 3:**
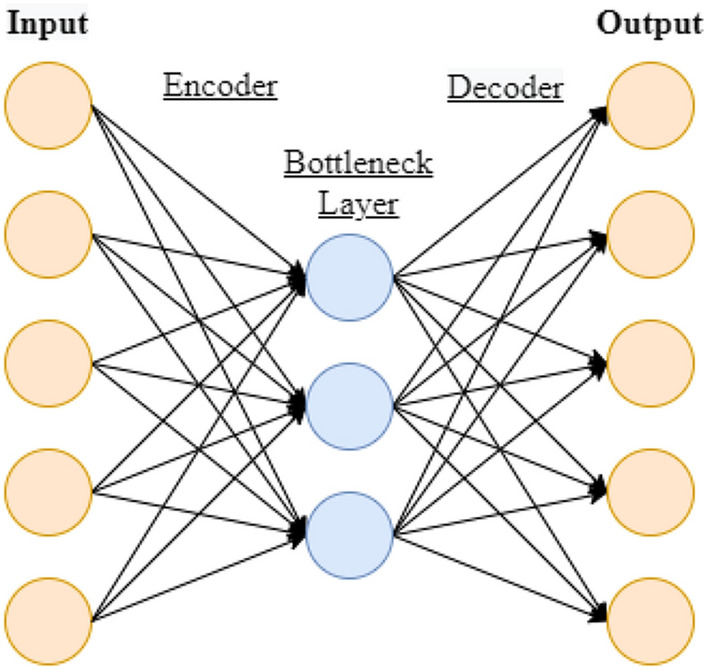
Standard Autoencoder Structure

Figure 3 shows the typical structure of a standard autoencoder. The encoder compresses the input data into a smaller dimensional space while the decoder reconstructs the input back into the original space. In particular, the encoder is also useful for compressing data for classification and regression tasks [55]. The decoding layers typically mirror the shape of the encoding layers. The autoencoder is trained through backpropagation, where a loss function is minimized. If the input dataset values are between 0 and 1, cross-entropy is taken as the loss function. In regression tasks, the minimum least squares loss function is used for training the ANN. Wang et al. [45] found that when the reduced dimension size is near the intrinsic dimensionality of the original data, prediction accuracy is high. In general, the number of bottleneck nodes is selected to be less than the input nodes to avoid overfitting and for mapping input nodes to a smaller dimensional representation. This is known as an under complete autoencoder. A penalty is applied to network during the reconstruction and the model learns the important attributes of the input data for decoding.

To ensure the latent attributes within a raw dataset are found, autoencoder models, incorporate regularization to enhance generalization capabilities depending on the nature of the dataset [56]. Denoising autoencoders resemble the standard autoencoder, except random Gaussian noise is introduced to the input dataset. The noise is applied to prevent the autoencoder from simply copying the input data to the output without learning features. These autoencoders aim to make the decoding layers resistive to small fluctuations of the input and can be stacked to initialize deep architectures [57]. The ‘denoised’ target output is rid of noise datapoints and has a different shape compared to the noise-filled input [58]. Consequently, the model learns a vector field that maps the input to a lower-dimensional manifold that accurately describes the noise-free dataset. Denoising autoencoders have been used extensively for improving clustering and statistic scoring techniques [59], 60], but have also been used in data processing in engineering design. Shang et al. [61] designed a convolutional denoising autoencoder for damage detection in bridges. The denoising encoder extracted features from field measurements that were sensitive to damage, but not noise. Contractive autoencoders make the encoder layers robust to small fluctuations in the input data. The model corresponds to the Frobenius norm of the Jacobian matrix of encoder layer activation functions. That is, the derivatives of the encoder hidden layer activations are minimized with respect to the input data. The loss function for contractive autoencoders penalize large derivatives in encoder layers; error is added when a small change in input leads to large changes in the encoding space. Sparse autoencoders introduce the bottleneck layer without reducing the number of nodes in the hidden layers. Rather than applying regularization to the weights of the network, sparse autoencoders apply penalties to the activation functions. Only non-zero nodes are activated. Thus the sparse autoencoder selectively activates regions of the network rather than every observation. The sparsity constraint is imposed either using L1 regularization or Kullback–Leibler-divergence (KL-divergence). L1 regularization adds the absolute value of the magnitude of penalty term coefficients to the loss function. KL-divergence considers the difference between two probability distributions. A sparsity parameter denotes the average activation of one neuron over the sample data set. By measuring the average activation of a neuron over its sample set, the model activates the neuron for a specific subset of observations. Variational autoencoders provide a probabilistic method for describing data in a latent space [62]. These autoencoders are applied in image processing and convert input datapoints into a probability distribution with weights and biases. Pu et al. [63] designed a variational autoencoder for deep learning applied to classifying images, labels, and captions. A CNN was used as the encoder to distribute the latent features to be decoded using a deep generative deconvolutional network.

Indeed, the autoencoder is a popular non-linear DR method for identifying and maintaining the key information of a dataset and generating a lower-dimensional latent space representation. However, the data used to train the autoencoder must resemble the testing data, otherwise, the autoencoder will decrease the performance of the classification or regression task on the reduced data afterwards. Furthermore, autoencoders determine the size of the latent dimension based on quantity of information rather than relevance. If the most relevant information of a dataset can be represented by a small portion of the input parameters, the autoencoder may eliminate a large amount of information leading to poor mappings and interpretations. Recent efforts have been made to improve upon the interpretability of autoencoder latent data representations [64], but require further research efforts for data containing high levels of non-linearity.

### Manifold Learning Methods

Manifold learning methods are unsupervised, non-linear dimensionality reduction techniques that project high-dimensional data onto a 2-D shape, or manifold. The method assumes the raw data lies on low-dimensional manifolds embedded within the high-dimensional space. Several manifold learning methods are present in the literature, such as locally linear embedding (LLE), iso maps, diffusion maps, and t-SNE.

#### Local Linear Embedding

Local linear embedding (LLE) produces low-dimensional embedding of raw data by relating training instances to its closest neighbor without involving local minima. The distances within local neighborhoods are preserved and nonlinear structures in the dataset are discovered. Given a training set of n-dimensional instances ${x}^{(i)}$, LLE first finds the k nearest neighbors based on Euclidean distances and expresses the instances as a weighted linear function. Equation (18) indicates the minimum least squares (MLS) cost function to be minimized to determine the weights [65].

18 $$\mathop \sum \limits_{i = 1}^{m} \left( {x^{i} - \mathop \sum \limits_{j = 1}^{m} w_{i,\;j} x^{j} } \right)^{2}$$

Once the weights ${w}_{i}{,}_{j}$ have been determined, the training instances are mapped to a lower dimensional vector ${y}^{(i)}$ with dimension $d$ such that $d<n$. Using the calculated weights from (16), ${y}^{(i)}$ and its dimensionality can be determined by minimizing another cost function as shown by (19).

19 $$\mathop \sum \limits_{i = 1}^{m} \left( {y^{i} - \mathop \sum \limits_{j = 1}^{m} w_{i,\;j} y^{i} } \right)^{2}$$

LLE offers many advantages as a non-linear dimensionality reduction tool [66]. Heureux et al. [67] used LLE to generate a low-dimensional representation of chemical data for simplifying quantitative structure–activity relationship methods. Compared to other non-linear dimensionality reduction algorithms, LLE proved to provide a stable representation that captured non-linearities in the data.

In LLE, the local distances of the data in high-dimensional space are preserved and the simple computations are fast and require little computation. However, the k-nearest neighbors search is based on Euclidean distances, which will fail when two points that do not lie in the same locally linear patch are grouped as neighbors. This makes LLE very sensitive to outliers and non-smooth manifolds. Furthermore, the assumption that datapoints lie on the manifold is not valid for all datasets and must be carefully observed in multi-class classification problems. Finally, when the number of neighbors $k$ is greater than the input dimensionality, the regularization in error functions becomes rank-deficient and causes problems in the LLE algorithm [68]. Thus, other variations of LLE have been developed to overcome the identified deficiencies. A modified version of LLE (MLLE) applies multiple weight vectors in each neighborhood, which overcomes the regularization problem at the cost of additional complexity. Wang et al. [69] developed the Maximal Linear Embedding (MLE) method which utilized geometric properties of Landmarks-based Global Alignment to create an isometric embedding of the manifold. Compared to the standard LLE method, MLE was capable of modeling the underlying modes of variability in the manifold and was more computationally efficient as the method is non-iterative. Other notable improvements upon LLE, such as robustness to noise and incorporating output for supervised learning, can be found in [70], 71, 72, 73]

#### Isomaps

**Fig. 4:**
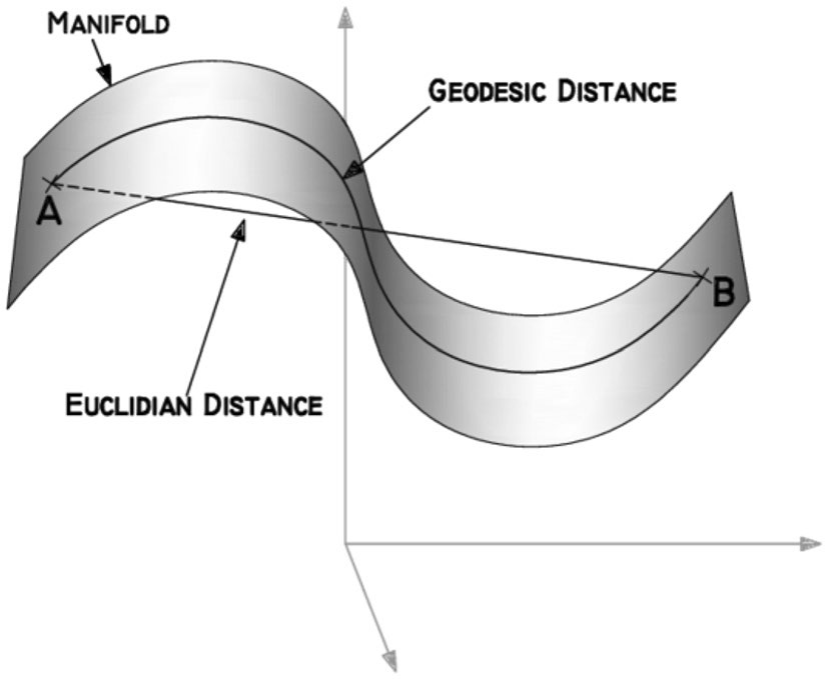
Euclidean vs Geodesic Distance on a manifold [162]

Isomaps [74], an extension of MDS, perform low-dimensionality projection assuming a linear local feature space formed by the nearest neighbors and that the global non-linear transformation to a lower dimensional space can be found by connecting the piece-wise local linear spaces. Unlike LLE and multidimensional scaling methods which maintain the Euclidean distances in the original feature space, isomaps preserve the geodesic distances between points along a manifold, as shown in Fig. 4. Constructing the transformation based on geodesic distances is beneficial for non-linear manifolds.

Like LLE, the procedure for constructing an isomap begins with constructing a neighborhood graph based on geodesic distances between nearest neighbors. The shortest paths of geodesic distances are commonly computed using Dijkstra’s algorithm and stored in a similarity matrix. After squaring the distance and double centering the similarity matrix, the eigendecomposition selects the first *K* eigenvectors corresponding to the highest eigenvalues, where *K* is less than the original dimensionality *n*. This is analogous to PCA. Xing et al. [75] applied isomaps to reduce the output space dimension of spatial/spatio-temporal fields as multi-variable functions. Correlation in the output data was exploited for the reduction of the permissible output space. Bhattacharjee and Matous [76] combined isomaps with NNs to create a reduced order model for multiscale analysis of heterogeneous hyperelastic materials. The macroscopic loading parameters are connected to a reduced space through the NN and the multiscale solution’s homogenization and localization were achieved.

Although isomaps accurately approximate distances in non-linear manifolds, they are not robust to noise perturbation and have a set of mathematical drawbacks. However, the similarity matrix may not always be positive definite, which is required for the projected manifold to be a Euclidean space. Choi and Choi [77] developed the kernel isomap method, which added robustness for non-Euclidean dissimilarity matrices. Next, the constructed matrix of geodesic distances must not have missing entries, otherwise the eigen-decomposition will fail [68]. Consequently, the neighborhood graph construction can be difficult, and the outliers lead isomaps to produce poor results. Several studies exist in the literature which extend upon the traditional isomap methods for improved pattern and image classification [78–80].

#### Diffusion Maps

Diffusion maps [81] achieve non-linear dimensionality reduction by re-organizing data according to parameters of its underlying geometry. A time-dependent diffusion process is created using the measured connectivity of the data set and the local geometry reveals geometric structures of the data set at different scales. The connectivity between two datapoints is determined by the random walk at these points expressed with a diffusion kernel function. The data is embedded in a lower-dimensional space, where the Euclidean distance between points in the reduced space is used to estimate the diffusion distance in the original domain. Unlike isomaps which preserve geodesic distances, diffusion maps are robust against noise, as it accumulates over all possible paths of a time step between points. The basic diffusion process involves four steps. First, a kernel is defined for creating the kernel matrix. Afterwards, the kernel matrix is row-normalized to generate a diffusion matrix, from which eigenvectors are then determined. Finally, the input vector is mapped to a lower-dimension diffusion space using the dominant eigenvalues and eigenvectors.

#### t-Distributed Stochastic Neighbor Embedding

t-Distributed stochastic neighbor embedding (t-SNE) is a non-linear dimensionality reduction method that has been extensively applied to image processing and speech recognition. The method was developed by [82, 83] for data visualization in lower dimensions. This probabilistic technique calculates the conditional probability of similar points, under a Gaussian distribution, in the high-dimensional space. A student t-distribution, or Cauchy distribution, with one degree of freedom, is often used to obtain a second set of conditional probabilities of similar datapoints in the low-dimensional space. The Cauchy distribution is chosen because it improves modeling for points that are farther apart in comparison to the Gaussian distribution. To ensure the probabilities in the high dimensional space are reflected as closely as possible in the low dimensional one, the difference between them must be minimized. The difference between these distributions is given by the KL-divergence. The algorithm minimizes the sum of KL-divergence of the datapoints using a gradient descent method.

The t-SNE method has extensively been applied to reducing dimensionality of hyperspectral data and data visualization on lower-dimensional planes [82, 83]. For instance, [84] used t-SNE for reducing the number of band information in hyperspectral imaging data for efficient processing. Non-linear similarity features between spectra were extracted and scaled into a 2D representation, which showed improved clustering quality compared with PCA. Pouyet [85] also used t-SNE for reducing hyperspectral data of paint pigments for 2D visualization.

t-SNE preserves the distances between nearby datapoints and performs well for data lying on non-linear manifolds compared to linear methods such as PCA. However, limitations exist for the t-SNE algorithm. Due to the heavy tails consisting of a large portion of probability mass in the Cauchy distribution, the t-SNE method cannot be extrapolated to reducing the data to more than three dimensions while maintaining the local data structure. Also, due to the non-convexity of the t-SNE cost function, the same choice of optimization parameters yields slightly different solutions each time t-SNE is performed. Thus, t-SNE produces a low-dimensional visualization of high-dimensional data but can lead to local optima due to the nature of the cost function to be minimized. Hsu and Huang [86] integrated t-SNE with distance hierarchy for dimensionality reduction of mixed datasets. Their proposed method preserved semantic similarities between categorical values and proved to be an improvement from traditional 1-of-k coding schemes for handling categorical data.

## Surrogate Modeling Methods

In engineering design, surrogate models/metamodels are employed for evaluating high-fidelity problems which would otherwise be too computationally expensive to analyze in its given dimensionality. Surrogate models have been implemented in Finite Element problems involving a large multitude of design parameters and constraints, such as rotor dynamics [87] and aerodynamics optimization.

Several surrogate models have been explored in the literature, such as Kriging, support vector machines (SVM), ANNs, radial basis functions, and polynomial response surface models (PSRMs). These can be separated into interpolating and non-interpolating models. Interpolation-based methods offer higher flexibility, while non-interpolating methods generate results with better interpretability. Due to the variety of engineering problems that surrogate models can be applied, selecting the appropriate model to implement typically depends on the available dataset size and number of parameters.

### Interpolation-Based Methods

Interpolation methods such as radial basis functions and Kriging calculate predictions based on surrounding data points. These methods are very flexible due to the variety of kernel choices and accurately predict unknown points in the vicinity of the data used to train the model.

#### Linear Interpolation

Radial Basis Function (RBF) surrogate models generate an interpolation function as a linear combination of basis functions. An interpolation function is developed for each training point and the coefficients of RBF are computed in the training phase. Equation (18) outlines the RBF prediction method, where ${w}_{p}$ and $w_{r}$ are vectors of polynomial and RBF coefficients, respectively, with $p\left(x\right)$ and $\phi \left( {x,xt_{i} } \right)$ vector mapping functions to the prediction $y$.

20 $$y = p\left( x \right)w_{p} + \mathop \sum \limits_{i}^{nt} \phi \left( {x,xt_{i} } \right)w_{r}$$

The inverse-distance weighting method interpolates unknown points based on weight averages of sample data. Equation (19) shows the inverse-distance weighting prediction equation. Within the weighting function $B\left(x,x{t}_{i}\right)$ in (20), $p$ denotes the order of the approximation. The order must be greater than 1 for continuous derivatives in the estimation.

21 $$y = \left\{ {\begin{array}{*{20}c} {\mathop \sum \limits_{i}^{nt} B\left( {x,xt_{i} } \right)yt_{i} } & {{\text{if}}\;x \ne xt_{i} } \\ {yt_{i}\quad\quad\quad \quad\;\;\;\;} & {{\text{if}}\;x = xt_{i} } \\ \end{array} } \right.$$

22 $$B\left( {x,xt_{i} } \right) = \left| {x_{i} - x_{j} } \right|^{ - p}$$

The Regularized minimum-energy tensor-product splines (RMT) method interpolates low-dimensional problems, with large datasets, by calculating spline coefficients based on minimizing the energy function. Similarly, the least-squares approximation method fits a linear model by minimizing the residual sum of squares between predicted responses and labelled responses. The second-order polynomial regression method utilizes a vector function of polynomial coefficients as the weighting function.

#### Kriging

Kriging, also known as Gaussian process regression (GPR), is a geostatistical method that generates an estimation surface based on spatial features in the sample data [88]. Unlike traditional interpolation methods, Kriging predictions include autocorrelation for measuring the accuracy of the predictions. GPR was first introduced in surrogate modeling for uncertainty quantification (UQ). These methods have been explored for UQ in groundwater flow applications [89–91] and QT interval estimation for preventing sudden cardiac death [92].

The Kriging space is assumed to be stationary with isotropic data. In addition to performing the spatial predictions through interpolation, this nonparametric Bayesian regression method also measures the interpolation accuracy and variance. This surrogate modeling method is based on the regionalized variable theory (RVT) which interpolates data and measures drift aspects of spatial properties under the assumption of stochastic characteristics in the dataset. In simple applications, RVT is defined by Matheron’s Intrinsic Hypothesis assumption [88], which assumes a constant local mean and stationary variance between points separated by a known distance. However, if a trend is observed in the random field, these assumptions are invalid, and the observed drift must be accounted for when applying the kriging method.

The Kriging method typically requires datasets with a moderate number of samples and low dimensionality for constructing reliable semivariograms. Furthermore, Kriging works well for problem sets where small changes in the input parameters lead to small changes in output. Predictions are made based on correlation variation, uncorrelation variation, and data trends. Fundamentally, the Kriging method predicts function values based on weighted averages of known values/data points near the point of interest and covariance functions of the data field. The covariance function acts as the kernel/weight of the Gaussian process to be optimized.

First, the Kriging method investigates the spatial variance of the dataset variables before constructing a function describing the degree of spatial dependence of the random field as a function of distance between points called an empirical semivariogram. Equation (23) describes the method for calculating semivariance $\gamma$, where *h* is the distance between *N* points located at *z*.

23 $$\gamma = \frac{1}{2N\left( h \right)}\mathop \sum \limits_{N(h)} \left( {z_{i} - z_{j} } \right) ^{2}$$

However, the semivariogram function is an empirical estimate of the variance and may not be positive definite; this is required for the final Kriging step involving matrix inverses. Different semivariogram models are designed to fit different types of phenomena and can be expressed as bounded and unbounded functions. Empirical semivariograms with an upwards concaving behavior can be modelled with unbounded power functions. Furthermore, this may be an indication of local drift or global trend in the random field, which must be eliminated to separate the deterministic and stochastic elements of variation. Duarte et al. [93] critically reviewed current methods for separation in the literature.

**Fig. 5:**
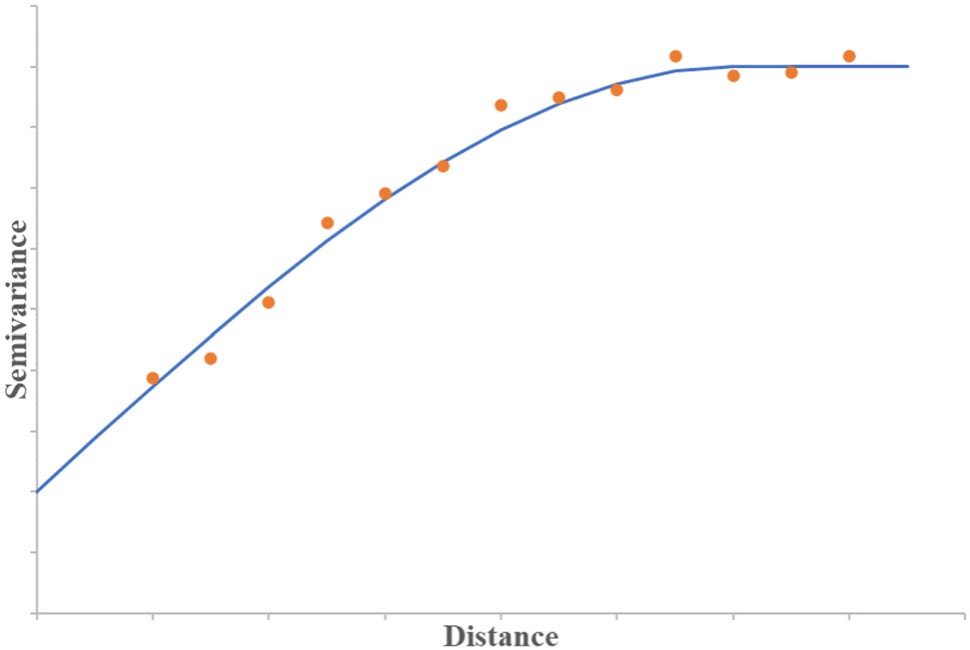
Spherical Semivariogram Model

If the semivariogram shows downward concaving behavior, bounded models such as spherical, exponential, Gaussian, and linear models are employed. For example, the spherical model shown in Fig. 5 is applied for progressive decrease in spatial autocorrelation with distance between data points.

As previously mentioned, the semivariogram describes the spatial autocorrelation in the dataset points. Semivariance is inversely proportional to correlation, thus as the distance between datapoints increases, the correlation between them decreases.

**Fig. 6:**
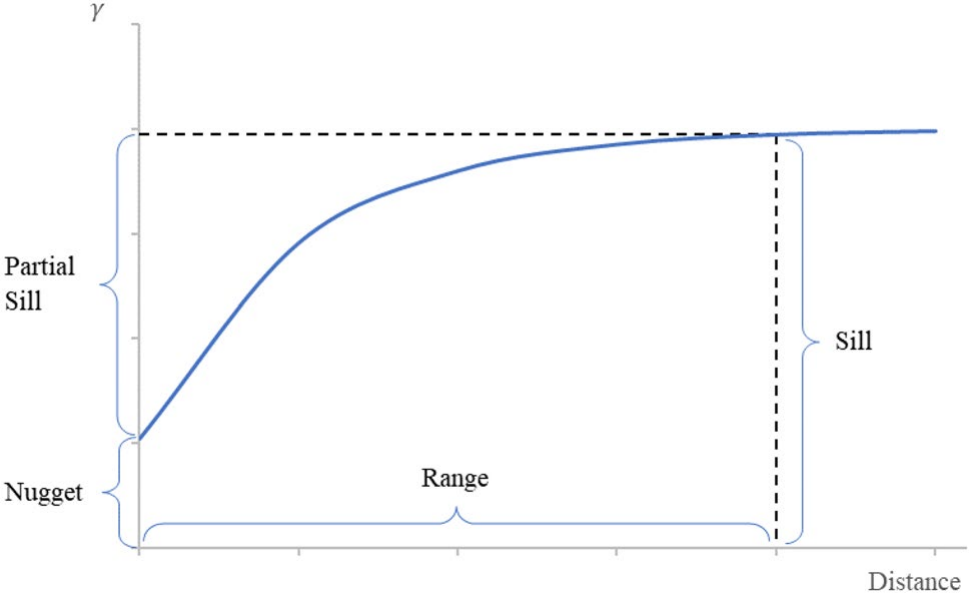
Aspects of Semivariogram

Figure 6 shows the different aspects of the semivariogram that define the spatial inferences of the random field. The sill is achieved once the semivariogram reaches the model range. Theoretically, at zero distance, the semivariogram measures zero semivariance or perfect correlation. However, measurement errors or spatial variations at distances smaller than the sampling interval may occur, which are represented by a nugget variance.

Upon fitting the data into a semivariogram model, kriging is applied to determine the Gaussian process weights/kernels based on minimizing the variance of the model.

The kriging can be classified into linear and non-linear methods. Geostatistical data can be described as shown in (22), where *Z* is the variable of interest, μ is the deterministic trend and ɛ are the autocorrelated errors.

24 $$Z\left( s \right) = \mu \left( s \right) + \varepsilon \left( s \right)$$

Simple kriging is the most general form of linear kriging methods. This method assumes *μ* and *ɛ* are known constants and the prediction relies upon the computed covariance function of the random field. In the absence of bias, the Kriging weights $\lambda$ are determined by (23), where *K* is the matrix of covariances computed by the selected semivariogram model, and *k* is the vector of covariances between the unsampled data point and surrounding datapoints. This is analogous to linear regression methods.

25 $$K\lambda = k = > \lambda = K^{ - 1} k$$

Ordinary kriging assumes $\mu$ is an unknown constant and is well-suited for interpolation when the random field remains locally stationary. While the method can be used when a trend is observed, the results cannot be analyzed to determine whether any data patterns occur due to autocorrelation or the identified trend in the random field. The universal kriging method assumes an overriding trend in data and models $\upmu$ as a polynomial. The polynomial estimation is subtracted from the original dataset to obtain the autocorrelation errors as random values. This method is suitable for random field variations consisting of drift and random components and can only be applied to data fields exhibiting a trend. Indicator kriging transforms the variable of interest, *Z(s)*, into a binary variable *I(s)* for classification problems. The disjunctive kriging method assumes the random field exhibits a bivariate distribution and assumes an unknown constant $\upmu$ and correlation coefficients as a function of distance between data points. To apply the bivariate model, the random field Z(s) is transformed into a normally distributed field *I(s)*. Unlike ordinary kriging which predicts the random field *Z(s)*, disjunctive kriging predicts a function of *Z(s)* by expanding into Hermite polynomials. Rivoirard [94] describes disjunctive kriging and nonlinear geostatistics in detail.

Kriging is a powerful geostatistical model for solving mapping relations between input and output. The method can further predict outputs of unknown values based on datapoints in the vicinity of the value of interest. While Kriging performs well for datasets with low dimensionality and moderate number of observations, its application is limited to isotropic datasets and are not useful for higher complexity models. Furthermore, Kriging experiences numerical stability problems when the sample points are too close together and computation cost grows proportionally with the size of the training dataset. Finally, if the number of observations is small, Kriging leads to poor, underfitted spatial representation and interpolation [95]. Hengl [95] proposed using Kriging regression methods for datasets with more than 50 observations with at least 10 observations per feature. Dimensionality reduction can be implemented to the original dataset to reduce the problem size for improved Kriging method-based results. In general, interpolation-based surrogate models are popular for their flexibility and accuracy of prediction, but these can lead to complex nonconvex formulations and mathematical instabilities.

Gradient-based surrogate models are also popular, as the consideration of derivative functions improves the prediction accuracy under the assumption of continuous gradients in the sample data. Lockwood and Anitescu [96] developed a gradient-enhanced universal kriging (GEUK) model for uncertainty propagation in nuclear engineering systems with limited available sampling data. Here, the gradient information was considered in the covariance matrix computations, which consisted of covariances between function observations and their associated derivatives. Compared to gradient-free approaches, the GEUK model required less computation and significantly improved prediction accuracy with the advantage of providing statistical prediction error estimates. Gradient-enhanced kriging surrogate models have also been used in biomechanics. Ulaganathan et al. [97] also considered gradient information in the correlation matrix. They tested their algorithm for predicting the wall thickness along the length of a simulated artery and found the gradient-enhanced kriging method achieved similar accuracy with 60% less training compared to standard ordinary Kriging. Indeed, the inclusion of gradient reduces the amount of computation required in Kriging. However, standard gradient-enhanced Kriging methods do not scale well with the dataset size, as the correlation matrix rapidly grows with additional sampling points [98]. The gradient-enhanced KPLS (GEKPLS) generates approximations around each sampling point using a first-order Taylor series. PLS is applied to different sampling points several times, where each PLS component determines the contribution of each variable near the sampling points. The global influence of each variable is computed by taking an average of all PLS hyperparameters and the Kriging is performed based on the reduced dimensional data. By reducing the number of hyperparameters and incorporating gradient computations, GEKPLS allows kriging to be applicable to high-dimensional problems.

Gradient-based methods can also be implemented into ANNs. Gradient-enhanced neural network (GENNs) apply gradient information during the training phase for regression problems. Compared to standard ANNs, which only minimize the error function, GENNs also optimize for the prediction error using partial derivatives. GENNs outperform standard ANNs for low amounts of training points, but can only be applied to synthetic datasets generated by physics-based models with continuous responses and defined gradients [98]. Bouhlel et al. [99] trained a GENN for airfoil design optimization in subsonic and transonic operating conditions. A modified version of the Sobolev method was used to train the ANN (mSANN), where gradient information was gradually introduced into the loss function. The model performance was evaluated based on the L1 and L2-norm of relative error of test and validation datasets in both operating conditions. The algorithm performed the optimization in the order of seconds, in comparison to CFD-based optimization tools which require hours. Compared to standard ANNs, the mSANN method showed 3% improvement based on the L2-norm of relative error. While gradient-based methods offer improvements to surrogate model predictions, they require gradient information which is not readily available in many datasets.

### Non-interpolation Methods

Non-interpolating surrogate models achieve the mapping between input and output by minimizing the error between a predefined function and the dataset. These methods tend to produce interpretable low-dimensional representations but lack the flexibility of interpretation-based methods when dealing with highly nonlinear data. Nevertheless, the presented methods have been used extensively as surrogates to large-scale models in various applications due to the simplicity achieved through the optimizations.

#### Support Vector Machines

**Fig. 7:**
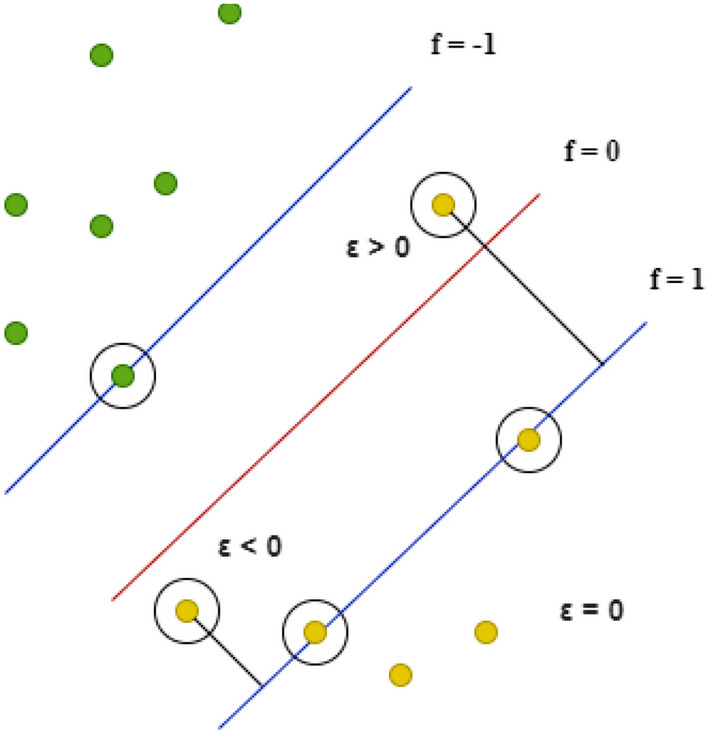
Middle line is the hyperplane. The circled points are support vectors. Errors in classification are evident and contribute to the hinge loss function

Several material sciences studies have employed support vector machines (SVMs) for binary classification tasks such as crystal structure identification [100] and material type classifications as a preprocessing step for regression [101]. Figure 7 shows the basic working principles of SVMs. SVMs aim to identify the optimal hyperplane (middle line) with maximum distance $\varepsilon$ from key datapoints called support vectors (circled points on outside lines). The method is based on geometric properties of the input data and outperforms other linear classifiers for small datasets. As shown in (24) and (25), the $\varepsilon$-insensitive hinge loss function [102] is defined as a function of weights/kernels, $K({x}_{i},x)$, and biases, *b*, to be adjusted during the training phase. Here, $f\left(x\right)$ denotes the SVM prediction and the contributed loss is determined based on the difference between the prediction error and threshold $\varepsilon$. The values ${\alpha }_{i}$ are hyperparameters that control the penalty applied for misclassifications.

26 $$f\left( x \right) = \mathop \sum \limits_{i = 1}^{N} (\alpha_{i} - \alpha_{i}^{*} )K\left( {x_{i} ,x} \right) + b$$

27 $${\text{Loss }} = \left\{ {\begin{array}{*{20}c} {0\quad \quad \quad \quad \;\,} \\ {\left| {y - f\left( x \right)} \right| - \varepsilon } \\ \end{array} } \right.\begin{array}{*{20}c} {{\text{if}} \;\varepsilon > \left| {y - f\left( x \right)} \right|} \\ {{\text{otherwise}}\quad \quad \;\;} \\ \end{array}$$

**Fig. 8:**
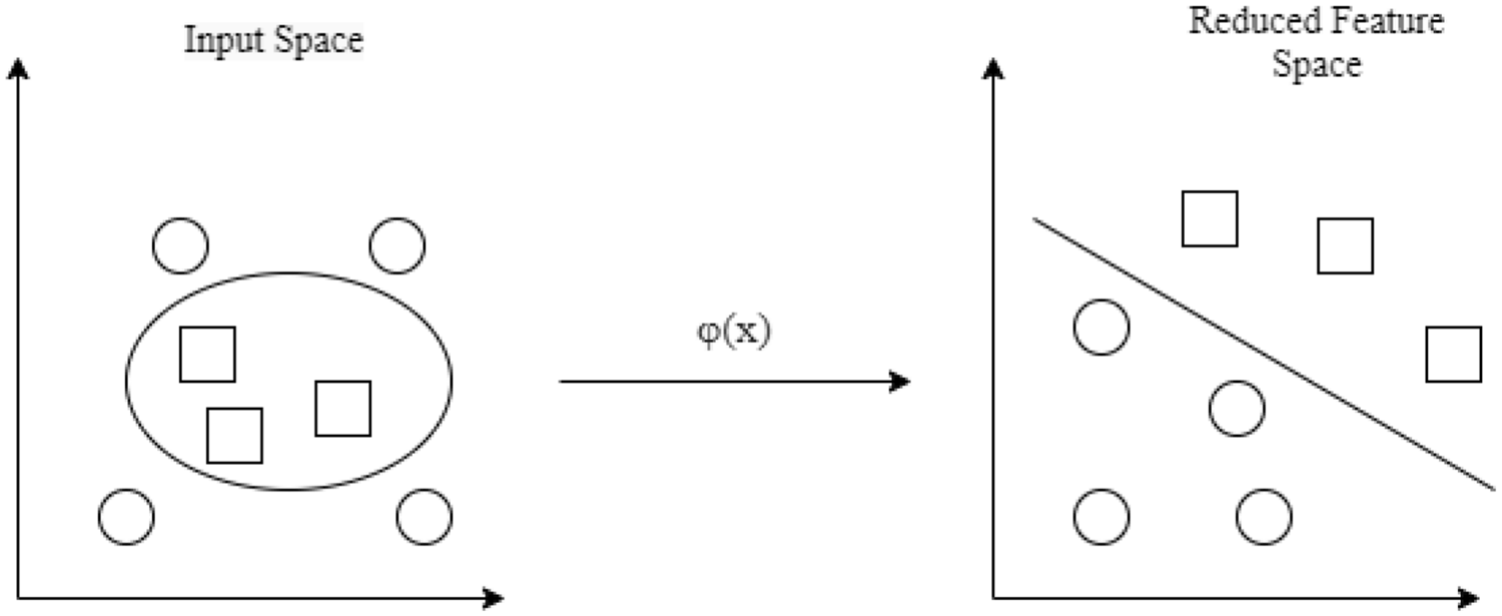
Non-linear kernel converts the input space to a higher-dimensional feature space for linear SVM

The kernel function can be selected to classify data that is not linearly separable. Polynomial or radial basis function kernels can be used to project the data into a higher-dimensional feature space, as shown in Fig. 8. The linear SVM is performed in this higher-dimensional feature space. This is similar to the procedure for kernel PCA. Again, the difficult problem of kernel parameter selection is prevalent for non-linear SVM.

#### Artificial Neural Networks

Artificial Neural Networks (ANNs) are multi-layer algorithms, based on the biological learning process of the human brain, that perform high-complexity regression and non-linear classification analyses. ANNs have been employed as data-fitting surrogate models for complex system output prediction [103, 104]. Papadopoulos et al. [103] developed a NN-based surrogate model for carbon nanotubes (CNTs) with geometric nonlinearities. An NN-based equivalent beam element was used for predicting the nonlinear behavior of CNTs at the nanoscale. The beam end-section displacements were used as inputs to the surrogate model with predicted output reaction forces. ANNs are also commonly used for multi-fidelity models. Zhang [105] developed a multi-fidelity deep NN (MFDNN) surrogate model for aerodynamic shape optimization of an aircraft. Their proposed MFDNN first evaluated the low-fidelity data before approximating the correlation with high-fidelity data obtained with a finer mesh. In the optimization framework, the current optimum solution of surrogate modeling was added to the high-fidelity database to obtain a better solution during local refinement. The method was compared with co-kriging and showed improvement in identifying discontinuities and nonlinearities during the optimization. Minisci and Vasile [106] developed an ANN for correcting aerodynamic forces in a low-fidelity models. The low-fidelity model generated samples globally over the range of design parameters, while the high-fidelity one locally refined the ANN in the latter optimization stages.

**Fig. 9:**
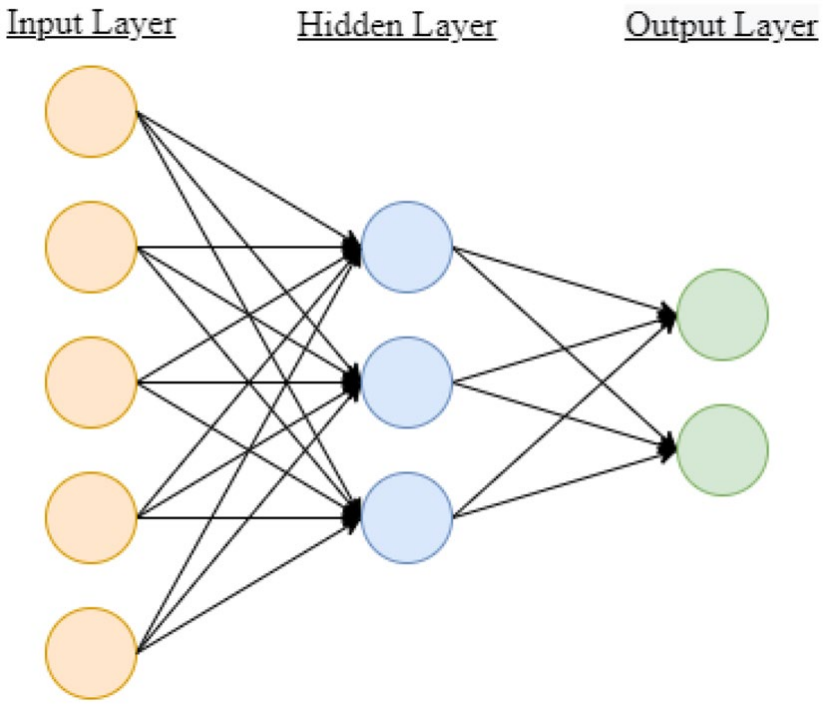
Structure of an Artificial Neural Network

The ANN’s popularity stems from its ability to identify complex mapping functions between input features and outputs. Figure 9 shows the general structure of the ANN with one hidden layer. Typical ANNs contain one input and output layer with one or more hidden layers. The number of nodes in the input *N* and output layers *M* is dependent on the number of dataset features and labels, respectively. The number of hidden layers and number of nodes *Q* is chosen based on the complexity and size of the problem. A smaller number of hidden nodes may cause the model to predict outputs with poor accuracy, while too many nodes may lead to overfitting the training data, leading to poor generalization of new data samples. Several methods and good practices have been developed for selecting the number of hidden nodes. Generally, the rule-of-thumb is for the number of hidden nodes to be between the number of input and output nodes. Heuristic and systematic methods have been developed for selecting the number or nodes and hidden layers [107].

The dataset is pre-processed prior to training the neural network. Normalizing the input features improves convergence capabilities of the ANN and the dimensionality of the dataset can be reduced to avoid long training times.

For a given sample, an input feature vector *X* is passed into the input layer of the ANN. An activation function φ transforms the *ith* input entry, with weights ${w}_{i,j}$ and biases ${b}_{j}$ connected to the *jth* output node of vector *u,* as shown in (28). Another transformation is performed on the hidden layer vector values to generate the prediction vector *y*, described by (29).

28 $$u_{j} = \varphi_{1} \left( {\mathop \sum \limits_{i = 1}^{N} w_{i,j}^{1} x_{i} + b_{j}^{1} } \right)$$

29 $$y_{k} = \varphi_{2} \left( {\mathop \sum \limits_{l = 1}^{M} w_{1,k}^{1} u_{l} + b_{k}^{1} } \right)$$

The sigmoid activation function as shown in (30), is commonly chosen for formulating the loss function to be optimized through back-propagation.

30 $$\phi \left( z \right) = \frac{1}{{1 + e^{ - z} }}$$

For classification tasks, the softmax function, which transforms the hidden layer outputs into normally distributed values between 0 and 1 in the output layer, is commonly used.

Finally, the ANN is trained to minimize the cost function through an optimization algorithm. Stochastic Gradient Descent (SGD) is the most popular optimization method that computes functional gradients based on the ANN’s weights and biases. These weights and biases are updated through the SGD and new outputs are predicted. The training loop continues for a specified number of epochs, the number of times the entire training dataset is explored by the ANN, and the test dataset is used for validating the model’s accuracy with the optimized weights and biases.

Modular neural networks have also been employed as surrogate models for multi-objective optimization in hydrology applications [108, 109]. Consisting of a series of independent NNs, each module in the surrogate model is assigned a specific task and reduces a single large NN into smaller components for efficient training and neuron robustness [110]. Sreekanth and Datta[109] linked a modular NN with a multi-objective genetic algorithm for solving a pumping optimization problem. Two stages of training and optimization of the combined model were sufficient to obtain Pareto-optimal solutions to the pumping problem, which was greatly reduced compared to a global surrogate model.

While NNs have proven to be high-accuracy surrogate models of computationally expensive problems, the training portion can be time-consuming and costly for high-dimensionality input and output situations. Dimensionality methods can be applied to reduce the cost of training by reducing node vector size at different levels of the ANN.

### Projection-Based Methods

Projection-based surrogate modeling methods [111] exploit the low-dimensional behavior inherent in many high-dimensional dynamic systems. The state of the system is approximated by a trial subspace and the high-fidelity model is projected onto a test subspace resulting in a square, lower-dimensional representation. Projection-based surrogate models retain the underlying structure of the model, which is important for accurate representation of system state in dynamical systems. Many empirically-based approaches for generating trial and test subspaces exist in the literature such as proper orthogonal decomposition (POD) [112], Krylov subspace methods [113], and dynamic mode decomposition [114]. POD has been applied in mechanical engineering for reduced-order aeroelastic modeling [115] and convection–diffusion modeling of contaminant concentration [116]. Krylov subspaces are mainly used for approximating solutions to high-dimensional linear problems [117]. Several authors have investigated the use of these methods for creating sub-models in electrical engineering [118, 119] and fluid mechanics [120]. Although Krylov subspace and other projection-based methods are useful for approximating solutions to high-dimensional problems, they suffer from a phenomenon known as breakdown. This occurs when the method is unable to approximate the next iteration of the solution due to mathematical complications. These were reviewed in detail by [121]. Algorithms have been proposed for circumventing breakdown in.

## Dimensionality Reduction in Surrogate Modeling

Surrogate models suffer from the curse of dimensionality (Verleysen & Francois), where complexity and computational demand scale according to the feature size of the problem. As mentioned before, surrogate models are non-intrusive, computationally inexpensive substitutes that mimic the real model response within parameter bounds. However, these suffer from large cost in training or computation for high-dimensionality problems. Thus, dimensionality reduction methods have been combined with surrogate models for extending them to high-dimensional problems [122].

### Principal Component Analysis Surrogate Modeling

PCA has been introduced extensively for reducing computational cost of designing surrogate models. For instance, [123] applied PCA for confining and scaling the domain of a data-driven surrogate model for predicting responses of a rat-race coupler and transformer. Compared with conventional kriging and RBF models, the proposed PCA-based surrogate model method predicted responses with higher accuracy. Higdon et al. [124] applied PCA for non-probabilistic, dimensionality reduction in Gaussian processes for uncertainty quantification of material properties. PCA has also improved optimization of surrogate models in calibration [125, aerodynamics [126], 127], and air pollution [128].

As mentioned before, PCA’s applicability is limited to linearly separable datasets and projections onto a linear subspace. Thus, kPCA has been used extensively for enhancing surrogate models. For instance, kPCA has been combined with PCE in the material science field [129], GPR in high dimensionality reliability analysis [130], and metamodels for accelerating evolutionary optimization algorithms [131]. Bird [132] developed linear and non-linear dimensionality reduction-based surrogate models for predicting nodal stresses and coordinates of a compressor blade. They found that the stress space exhibited more nonlinear behavior than the coordinate space and found that kPCA and LLE resulted in the lowest surrogate error in high complex design spaces. While these kPCA-based DRSMs improved computation times and surrogate errors, there is a lack of research to determine the best general DRSM for a given dataset. The authors identify the need to test these algorithms on new datasets to determine their usefulness and applicability in other fields.

### Partial Least Squares Surrogate Modeling

PLS has also been used in combination with different surrogate modeling techniques. Straus and Skogestad [133] developed extended surrogate model fitting methods by incorporating PLS regression for reducing the number of independent variables in an ammonia process case study. The non-linear surrogate models were fitted to the PLS latent variables. The preprocessing with PLS improved the surrogate model fit by a factor of two. Ehre et al. [134] developed a polynomial chaos expansion (PCE) method based on PLS called PLS-PCE for global sensitivity analysis in high dimensions. The novel algorithm had comparable accuracy as Monte-Carlo-based solutions and approximations based on low-rank appoximations for two different numerical problems. The authors provided recommendations for further research into extending their method for general basis adaptation.

### Variational Autoencoder Surrogate Modeling

Variational autoencoders (VAEs) have been extensively used for feature extraction and data reduction in the preprocessing step for regression and classification tasks. Na et al. [135] used a variational autoencoder for reducing data input to a DNN for predicting CFD gas dispersion rates and death probability. The proposed algorithm optimized weights based on minimizing the mean squared error (MSE) and was compared against standard autoencoders and single-layer neural networks. The VAEDC-DNN predicted results with 48% less error on the probability of death and accurately predicted nonlinearities of image cracks and topography. Laubscher and Rousseau [136] combined multiple fully connected VAEs with a DNN for compressing CFD data of a simulated turbulent jet diffusion flame. The VAE and DNN were trained separately. The VAE was trained on the target data set, whereas the DNN was trained to predict latent layer encodings. Compared to a standalone DNN, the VAE-DNN yielded lower MAE and provided accurate predictions of 2D contours of fluid values in a jet flame combustion setup.

**Fig. 10:**
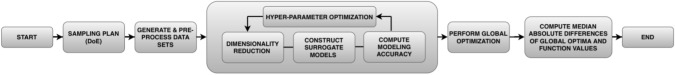
Hyperparameter training and optimization of DRSM from [137]

Ullah et al. [137] compared the performance of PCA, kPCA, autoencoders, and variational autoencoders combined with Kriging and Polynomial Regression. A total of 72 cases were explored, with their performance aggregated across ten benchmark functions. Their experimental set-up and hyperparameter optimization process are shown in Fig. 10.

The relative mean absolute error function (RMAE) was used to evaluate the quality of each low dimensionality surrogate model (LDSM). The hyperparameters were optimized for both DR and surrogate model together based on the aggregated quality of the LDSMs on all benchmark functions. Overall, their study showed that autoencoder-based LDSMs achieved the best modeling accuracy (lowest RMAE values) in 132/720 cases. With regards to global optimality, LDSMs based on PCA and kPCA performed better in combination with Kriging, while all LDSMS based on polynomial regression performed similarly in most cases. While this study provides insight on the performance of different LDSM methods, further research must be conducted using real-world data, as the benchmark functions explored were noiseless, single-objective functions. Further work must be performed to validate these models’ performance for problems exhibiting large uncertainties.

### Dimensionality Reduction for Support Vector Machines

SVM has shown to be robust against the curse of dimensionality in classification tasks [138]. Yet, several studies explore the use of DR methods as preprocessing in SVM classification, with the intent of improving classification accuracy and speed. George [139] applied PCA as preprocessing for SVM classification for anomaly detection. Their results showed a decrease in execution time and increased accuracy for classification. However, their conclusions require further validation using different anomaly datasets with higher number of features. Wang and Carreira-Perpinan[140] studied the influence of dimensionality reduction in SVM classification. They used auxillary coordinates to jointly optimize the classification error jointly over an RBF-based DR mapping and a wrapper approach to classification. The final non-linear low-dimensional classifier achieved similar errors as previous literature methods but accelerated model training time. Furthermore, their results showed the DR mapping eliminated variation in the original data space. Bai et al. [141] compared different DR methods, such as PCA, LLE, and Isomaps, for low-dimensional representation of data prior to developing an SVM modeling applied to manufacturing quality prediction. Based on qualitative and quantitative analysis, each DR method improved prediction accuracy compared to SVM alone. In particular, the Isomap-SVM model showed the best regression performance for the multi-parameter manufacturing system.

In terms of applicability, DR-based SVM classification methods have obtained state-of-the-art prediction accuracies in the medical field. Most commonly, LDA has been superior compared to other linear DR methods for SVM models in epilepsy prediction [142] and detection of diabetes disease [143]. Ali [144] extended these works and developed a threefold hybrid algorithm which includes a genetic algorithm optimization component with the LDA-SVM model. The algorithm was validated on the HCC dataset and improved prediction accuracy while lowering processing time in hyperparameter optimization and training time.

### Dimensionality Reduction for Manifold Learning

Manifold learning methods have been used for constructing surrogate models in the data’s intrinsic dimensionality for cost-efficient representation of high-dimensional systems. Kalogeris and Papadopoulos [145] used diffusion mapping for constructing locally clustered interpolation schemes between the parameter space, diffusion map space, and solution space for linear stochastic uncertainty qualification problems. Two case studies were conducted, and the probability density functions were predicted with 80% less time, while maintaining high accuracy, compared to full computation of the detailed models. Chen et al. [146] developed a manifold Gaussian process method using isomaps for dimensionality reduction. The method was applied to extract coupling coefficients in fourth and sixth order coupling filters, where few samples of high dimensional data were available. The test error of both filters was reduced with the isomap dimensionality reduction. Decker et al. combined POD, LLE, and isomaps with RBF for reduced-order modeling of hypersonics systems. The POD was unable to resolve discontinuous features without oscillatory behavior, but LLE and isomaps accurately predicted the steady-state response and resolved geometric discontinuities. However, this study was limited to simple 2D CFD cases and further research must be done considering shape variations and optimization of parameters in training LLE and isomaps. More recently, authors employed nonlinear manifold methods due to their ability to represent nonlinear field features for studying shocks [147, 148] and adaptive design of experiments [149]. Finally, t-SNE has become a standard for interpreting DNNs, revealing information about the data structure at local and global scales [150, 151]. Furthermore, the method generates better visualizations compared to other manifold learning methods [82, 83]. However, research that utilizes the t-SNE method has largely been limited to 2D and 3D representations.

### Dimensionality Reduction for Kriging

Gaussian process-based (GP) methods, such as Kriging, perform poorly in high-dimensionality problems. Thus, dimensionality reduction methods have been implemented for overcoming these issues.

Kriging Partial Least Squares (KPLS) [152] performs the Kriging after reducing the number of hyperparameters through PLS. Consequently, the KPLS method converges much faster than standard Kriging methods. An extension of KPLS is the kernel KPLS (KPLSK) method which consists of two steps. First, KPLS is performed to estimate the hyperparameters in a reduced space. The hyperparameters are transformed back into the original, higher dimension space where the estimated parameters are now used as a starting point for optimizing the standard Kriging interpolation procedure.

Constantine et al. [153] integrated the active subspace method with Kriging for constructing a response surface. The method was tested on an elliptic PDE model with 100 Gaussian random variables and proved to yield a more accurate approximation of the response surface compared to kriging on the full domain. PCA methods have also been applied to reduce computational burdens in Kriging. Steer et al. [154] developed a PCA-Kriging model for predicting FEA residual limb morphologies and prosthetic socket designs. The computation expense was reduced by an order of six magnitudes compared to traditional Kriging and produced real-time rendering of pressure and shear distribution of the residual limb, indicating the potential of surrogate models for improving clinical settings. However, this study simplified the dynamic load cases and total surface bearing design to three press points. Further studies in this field need to consider complex parametric socket models and variation in morphology and loading. Lataniotis et al. [25] proposed the supervised DSRM method, which optimized parameters through combined compression of kPCA with PCE and Kriging surrogate models and reduced dimensionality through solving a nested optimization problem. An anisotropic Gaussian kernel was used in both surrogate models and the DSRM was trained on three benchmark problems and compared against classical approaches of sequentially tuning the dimensionality reduction and surrogate model parameters separately. The DSRM showed superior performance based on generalization error, but still suffered from the curse of dimensionality in the nested optimization of objective functions. Gadd et al. [155] developed a surrogate modeling approach that combined a novel non-linear dimensionality reduction method, local tangent space alignment (LTSA), with GPR using Markov Chain Monte Carlo for UQ in groundwater flow models. In contrast to other manifold learning methods, LTSA is a local method which estimates points on the manifold on localized regions rather than identifying a global basis solution for the reduced space.

Other GP-based methods such as Bayesian surrogate models also require smooth patterns of objective functions to accurately represent full-scale systems. Lei et al. [156] developed a more flexible and adaptive Bayesian surrogate modelling method for improving search efficiency and robustness in two different case studies. By implementing Bayesian multivariate adaptive regression splines, the authors found that their proposed method required less trials and computation when performing material discovery compared to the standard GP-based methods. Moriconi et al. [157] utilized the response surface method for reconstructing the parameter space in a lower dimensional representation for improving Bayesian optimization of black-box functions. Compared to previous Bayesian optimization methods, their proposed method reduced computational complexity with faster computations when a small number of data points were available. Bayesian optimization has also been enhanced using a restricted-projection-pursuit (RPP) GP [158]. In their study, the authors used RPP to reduce the function dimensionality without restricting the projection to an axis-aligned representation. Compared to previous methods such as additive models and low-dimensional assumption models, the proposed method provided a more expressive framework for Bayesian optimization.

Linear and Kernel-based dimensionality reduction in Kriging and other GP-based surrogate models extend the method’s applicability to higher dimensions and has proven to increase accuracy of the method. However, the discussed methods are largely dependent on specific types of kernels and require further research to generate kernel-based Kriging models for general application.

### Dimensionality Reduction for Projection-Based Surrogate Models

PCA has been applied with Krylov subspace models for computationally efficient and inexpensive modeling of large-scale systems. Awais et al. [159] created a hybrid algorithm using PCA for dimensionality reduction and the Arnoldi algorithm for constructing an oblique Krylov subspace projection. Their hybrid algorithm was validated on random models with 100 and 200 input parameters. Compared to the standard scheme of using balanced truncation for reducing the model size from 200 to 10 dimensions, the hybrid method reduced computation time by 43% and floating-point operations per second (FLOPS) by 60%. However, optimizing the trade-off between reducing instabilities generated by oblique projection methods and improving computational time remains a challenge. Ubaru et al. [160] proposed a method for simultaneously estimating the underlying principal subspace dimensionality of covariance matrices and applying it to the Krylov subspace method to compute an approximation of the subspace. This method known as Krylov PCA uses the Lanczos algorithm for identifying the key eigenvalues and approximating the principal subspace of the covariance matrix. Since the covariance matrix for PCA does not need to be formed with this method, less storage space is required for the overall modeling process, with runtimes in the order of seconds to complete. While Krylov subspace DRSMs have been applied successfully for reducing computation times of large-scale systems, projection-based methods suffer from several convergence issues, which require further investigation to validate their use in other applications.

## Discussion

Table 1 summarizes the literature on combined dimensionality reduction-surrogate model (DRSM) methods. In the accuracy column, only the model structures with the best performance are reported. The literature can be visited for further details on training and optimizing the model structures for reducing error and computation. Overall, DR methods have benefitted surrogate models by reducing computation times and improving prediction accuracies.

**Table 1:** Summary of DRSM Literature

Ref	Year	Application/Dataset	DR Method	Surrogate Model	Comp. time (without DR) or computational complexity	Accuracy (criteria)
Koziel and Pietrenko-Dabrowska [123]	2020	Transformer and rat-race coupler model (RRC)	PCA	Kriging	Transformer: 2 min RRC: 6 min	Transformer: 97.9% on 800 samples (RMS) 93.9% without DR RRC: 99.1% on 800 samples (RMS) 92% without DR
Kamali et al. [125]	2007	Calibration of watershed	PCA	Kriging	–	37% less predictions outside threshold error with 40% less simulations (Nash–Sutcliffe Criteria [163])
Li et al. [164]	2021	Predictng SUR and DixonPrice functions	PCA	Kriging	SUR function: 216.5188 s with Kriging 14.1654 s with KPLS 12.8934 s with PCA-Kriging	SUR function RMSE: Kriging: 1.11E4 KPLS: 7.0E3 PCA-Kriging: 8.8E3
Tao et al. [126]	2019	Aerodynamics optimization	PCA	Deep Belief NN	–	3.11% compared with CFD lift-drag ratios results 1.08% compared with CFD drag coefficient predictions
Ma and Zabaras [129]	2011	Stochastic Analysis of material property variation in hetereogeneous media	kPCA	PCE	–	11.4% skewness error with kPCA compared to 100.6% with PCA 0.5% MSE on reconstruction
Bird [132]	2020	Stress on turbomachine compressor blade	PCA, kPCA, Isomap, LLE	RBF	Isomap-RBF: 14 min (hours with FEA) PCA-IT: 3 min	2.47% on Isomap-RBF nodal stresses (double-NRMSE) 0.7% on inverse-transform PCA (PCA-IT)
Straus and Skogestad [133]	2017	Ammonia Process	PLS	ANN	–	6 latent variables: (MAE) 0.025 on Pressure 0.02 on Temperature 0.4 on extent of reaction
Ehre et al. [134]	2020	Global sensitivity analysis on truss and plate	PLS	PCE	–	< 0.01 for over 1000 observations of 1^st^ critical plate stress (mean relative error)
Na et al. [135]	2018	Toxic gas release model	VAE with deep convolution layers	CNN	1 s (700 s with CFD model)	0.00246 (MSE) 47.7% lower than other models compared
Ullah et al. [137]	2020	Several benchmark functions	PCA, kPCA, Autoencoders, VAE	Kriging Polynomial Regression	–	PCA-Kriging had best accuracy for optimization AE-based surrogates had highest modeling accuracy
Laubscher and Rousseau [136]	2021	Turbulent jet diffusion	VAE	DNN	–	0.3% on species concentrations < 7% on temperature and velocity field (MAE)
George [139]	2012	KDD99 benchmark dataset	PCA	SVM	0.009 s (0.293 s)	93.75% (classification)
Bai et al. [141]	2018	Manufacturing quality prediction	PCA, LLE, Isomap	SVM	–	Isomap-SVM: 0.03 and 0.023 on two test cases (RMSE)
Subasi and Gursoy [142]	2010	EEG signal classification	PCA, LDA, ICA	SVM	–	LDA-SVM: 100% accuracy SVM: 98%
Calisir and Dogantekin [143]	2011	Diabetes classification	LDA	SVM	–	89.74% (9% higher than GRNN)
Ali et al. [144]	2020	Hepatocellular carcinoma prediction	LDA	RBF-SVM	Optimization time: 1.15 s (41.56 s)	90.3% (78% without LDA)
Kalogeris and Papadopoulos [145]	2019	2D Cantilever 3D wrench model	Diffusion Map	Polynomial interpolation	50 k runs: 876 s (46532 s with full model)	7.62% error in pdf (polynomial density function) compared with Monte Carlo
Chen et al. [146]	2020	Microwave components model optimization	Isomap	Gaussian Process	–	0.84% on 4^th^ order model (MAPE) 2.02% (DNN)
Franz et al. [147]	2015	Steady transonic flows	Isomap	RBF interpolation	*O(nm(mlogm* + *E))* E = number of edges M = number of snapshots N = full-space dimension	Order of 0.01 (relative error)
Njock et al. [151]	2020	Soil liquefaction prediction classification	t-SNE	Evolutionary NN	–	97% on testing 94% on training
Bouhlel et al. [152]	2018	Griewank function	kPLS	Kriging	20.49 s for 300 samples (94.31 s without kPLS)	0.99% error (1.39% without kPLS)
Constantine et al. [153]	2014	Elliptic PDE	Active subspace	Kriging	–	0.11 (relative to Monte Carlo)
Steer et al. [154]	2018	FEA residual limb morphologies	PCA	Kriging	1.6 ms (30 min for FEA convergence)	< 3% soft tissue strain < 4 kPa residuum tip pressure
Gadd et al. [155]	2018	2D Darcy’s Law with contaminant mass balance Richards equation in 3D	LTSA	Gaussian Process	O(m^2 N) N = number of design points m = inducing points	Similar to results from numerical simulator
Awais et al. [159]	2007	Random models with 100 and 200 parameters	PCA	Krylov Subspace	43% lower CPU time 60% lower FLOP time (both compared to BT method)	–
Ubaru et al. [160]	2019	Signal Detection	PCA	Krylov Subspace	Order of seconds (runtime)	–

PCA-based DRSMs [123], 125, 126, 127, 128, 132 have shown to improve modeling accuracies while reducing the number of samples required to complete the training portion of the surrogate model. Other researchers have found that applying kPCA for nonlinear DR captured skewness behavior better than linear PCA [129]. Manifold-based DR methods [145, 146], 147, 148] have mainly been used with interpolation methods and showed improvement in both computation time and prediction error compared to traditional Monte Carlo and NN models.

DR methods have improved SVM classification accuracies in manufacturing quality [141] and medical data [143]. Kriging DRSMs [152, 153], 154] have mainly been tested on benchmark functions and shown improvements compared to standalone surrogate models and Monte Carlo functions. Projection-based DR methods have also been applied for improving predictions using GP-based surrogate models [161]. Finally, PCA has been applied to Krylov subspace methods for reducing memory use and time required to obtain the lower-dimensional model of the data space [159], 160]. These methods, however, require further research to circumvent the convergence issues.

By identifying and retaining the key information in the data, DRSMs have successfully improved optimization and calibration methods in engineering fields such as aerodynamics and finite element analysis. However, DRSMs are a new field of research and have mainly been applied to datasets with minor nonlinearities. In fact, surrogate modelling methods such as random forest and tree -structured Parzen estimator have not been explored with dimensionality reduction methods. Several of the reviewed literatures propose the need to apply their methods to more complex cases to validate the DRSM methods for general use cases and explore ways to overcome the mathematical limitations imposed.

## Conclusion

Machine learning algorithms have largely been applied for aiding surrogate modeling construction and reducing computational costs of modeling full-scale, high-dimensionality problems. In this paper, we discussed the state-of-the-art methods of linear and nonlinear dimensionality reduction methods and surrogate modeling, as well as the literatures that combine them. DR methods have been used to create more cost-efficient surrogates. Traditional surrogate models perform poorly because of limitted data availability, high dimensionality, and rigid training. The literature shows the DR-SR methods have potential for other fields of application beyond their traditional use in quality assurance and uncertainty quantification. However, DRSM methods are a new field of research and require further exploration to validate their applicability to datasets with uncertainty and ambiguous underlying structures. Indeed, these methods are still evolving and will continue to be applied to high-dimensional problems in other engineering fields.

## Data Availability

Not applicable as this is a review paper.

## Appendix

Acronyms

**Table:**

DR	Dimensionality reduction
ML	Machine learning
DRSM	Dimensionality reduction-surrogate model
PCA	Principal component analysis
kPCA	Kernel PCA
LDA	Linear discriminant analysis
FA	Factor analysis
FAMD	Factor analysis of mixed data
MCA	Multiple correspondence analysis
PLS	Partial least squares
KPLS	Kernel PLS
GEKPLS	Gradient-enhanced kernel PLS
ASM	Active subspace methods
ANN	Artificial neural network
CNN	Convolutional neural network
RNN	Recursive neural network
GENN	Gradient-enhanced neural network
mSANN	Modified Sobolev artificial neural network
MFDNN	Multi-fidelity deep neural network
KL-divergence	Kullback–Leibler-divergence
LLE	Local linear embedding
MLLE	Modified local linear embedding
MLE	Maximal linear embedding
MDS	Multi-dimensional scaling
t-SNE	t-Distributed stochastic neighbor embedding
SVM	Support vector machine
PSRM	Polynomial response surface models
RBF	Radial basis function
RMT	Regularized minimum energy tensor product
GPR	Gaussian process regression
FLOP	Floating-point operations per second
UQ	Uncertainty quantification
RVT	Regionalized variable theory
SGD	Stochastic gradient descent
PCE	Polynomial chaos expansion
MSE	Mean squared error
MAE	Mean absolute error
MAPE	Mean absolute percentage error
GP	Gaussian Process
RPP	Restricted-projection pursuit
